# An effective range estimation and state-of-charge to mitigate range anxiety in electric vehicles

**DOI:** 10.1016/j.heliyon.2024.e41494

**Published:** 2024-12-26

**Authors:** Shana Lakshmi Prasad, Abhishek Gudipalli

**Affiliations:** School of Electrical Engineering, Vellore Institute of Technology, Vellore, India

**Keywords:** Drive cycle, Electric vehicle (EV), Energy consumption, Performance range, State-of-charge (SoC)

## Abstract

This article presents a performance investigation of the range and state of electric charge vehicles based on different drive cycles and analyzed. Range anxiety is the major concern in electric vehicles globally. To mitigate the range anxiety issue by reducing the energy consumption in electric vehicles further reduces the range-related problem, and the state of charge reduces the demand for driver's power. The drive cycles predict, optimize, and simulate the performance range of Ev's real-world driving conditions. This research thoroughly focuses on the performance range of electric vehicles incorporated with the state of charge based on various standard drive cycles. A drive cycle plays a vital role in estimating energy consumption (EC) per km. There are many standard drive cycles World Wide Harmonized Light Vehicles Test Procedure (WLTP Class-3), the New European Drive Cycle (NEDC), the Modified Indian Drive Cycle (MIDC), the Indian Drive Cycle (IDC) used to investigate and evaluate Electric vehicle (EVs) performance range and state of charge (SoC). Range obtained from above drive WLTP Class-3, NEDC, MIDC, and IDC drive cycles 135.1123.8115.7 and 101.5 km, respectively. Due to improved road conditions, the WLTP Class-3 drive cycle offers more than the others. Due to road conditions, the IDC drive cycle offers a shorter range of 101.5 km.

## 1 Introduction

Electric vehicles (EVs) have become the solution to air pollution and power consumption caused by traditional petrol and diesel vehicles due to their zero-emission, quiet operation, and high efficiency. However, EVs are limited by their limited range, lack of indigenous technology, long charging times, and high initial costs [1,2]. Drivers' main concerns regarding electric vehicles are the limited driving range and long charging times. This often leads to what is known as "range anxiety," where drivers worry about running out of battery power before reaching their destination. Range anxiety is a significant issue for drivers who are considering purchasing an electric vehicle, as it is caused by the technical limitations of EV batteries, which can make it difficult for drivers to fully embrace this new technology [[3], [4], [5]]. To reduce concerns about running out of power, it is crucial to have a large battery capacity, adequate charging network, quick charging times, and accurate calculations of energy consumption per kilometer along the planned route before starting the journey. EV technology changes daily, but battery mass and cost are important constraints in the adaptation process. Therefore, in an electric vehicle's design phase, accurate energy efficiency estimation has become critical in affecting battery cost, weight, and size [6]. Lithium-ion batteries are often used in electric vehicles (EVs) because they offer higher efficiency, energy density, specific energy, longer cycle life, and lighter weight than lead-acid batteries. Consequently, it is popularly known as the preferred choice for powering electric vehicles. When developing a battery system for an electric vehicle (EV), it is essential to accurately determine the energy consumption per kilometer (EC per km). To estimate, the driving wheels of the vehicle can be studied. Historically, drive cycles have been used in designing and developing internal combustion engines for conventional automobiles, requiring standard battery storage systems [7,8]. The energy storage capacity of battery cells used in electric vehicles (EVs) is low compared to conventional automobiles. It affects a range of electric vehicles. Range is the buyer's primary concern, so accurate driving cycle analysis is critical to minimizing the energy consumption of an electric vehicle (EV). Automakers provide greater distance than vehicles at a single charge at reduced costs. Energy consumption (EC) is a key area of study and development in transportation systems [9]. EC (energy consumption) in electric cars (EVs) is a key factor in the design and development of EVs, as the acceptance of EVs mainly emphasizes the vehicle's range. A more affordable starting price will attract a wider spectrum of consumers, leading them to opt for electric vehicles, However, to improve productivity and enhanced reliability digital twin technology overcomes the gap between real-world system integration the virtual performance but initial investment cost and data security is the major challenging in digital twin [10]. Further estimating the state of charge accurately requires an approach that is highly reliable and less computationally expensive [11].

**Fig. 1:**
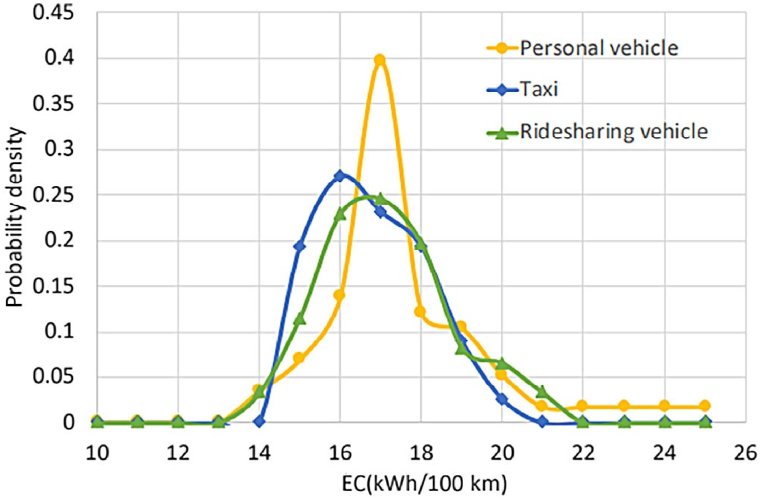
Three different vehicle types of energy consumption.

Seasonal effects on EC and driving range were studied, revealing a correlation between ambient temperature and EC, with low temperatures contributing to increased EC levels. This study highlights the impact of the environment on EC, as shown in Fig. 1. To investigate energy consumption (EC), it is important to establish the driving cycle and use efficient and scientific methods for this purpose. Markov chain and Monte Carlo simulation methods are utilized to construct the drive cycle. By using a real-world drive cycle, EC can be accurately predicted, which in turn can help determine the range of the vehicle [9]. The EV dynamic model is analyzed using MATLAB/Simulink to calculate the EC. Energy consumption is 15.82 kW-hours per 100 km, and the NEDC standard driving range is 177 km. WLTP estimates 157-km [12].

**Fig. 2:**
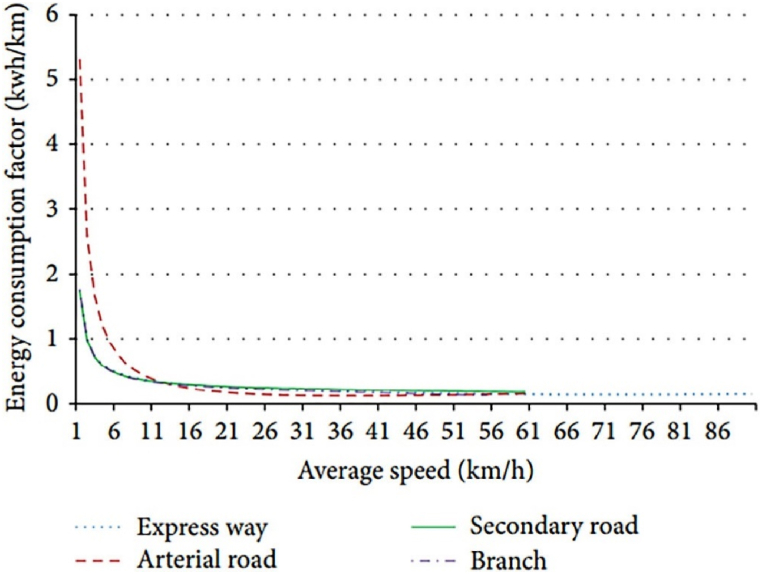
Energy consumption effect with various road types.

Battery utilization was presented, and the problems related to urban-scale EVs were discussed. An accurate estimation of EC was also done [13]. It is important to consider the type of road when estimating the EC (Energy consumption) for vehicle travel at a constant speed. Several studies have shown that the EC varies depending on the type of road. Expressways, secondary roads, and branch and arterial roads were analyzed to determine their respective ECs. The results showed that the EC of arterial roads is higher than that of the other three types of roads (as shown in Fig. 2.) [14].

An algorithm has been developed to estimate EC accurately by considering various parameters such as driving mode, environment conditions, traffic situation, and road contour information. Efficient energy consumption (EC) is predicted using online and offline algorithms. An offline algorithm is used at the starting point, while an online algorithm is used while driving [15].

An algorithm has been developed to estimate EC accurately by considering various parameters such as driving mode, environment conditions, traffic situation, and road contour information. Efficient energy consumption (EC) is predicted using online and offline algorithms. An offline algorithm is used at the starting point, while an online algorithm is used while driving [16].

Emphasized the need for precise EC using an application programming interface [17]. Several factors can affect the electrical efficiency of a vehicle, including driving habits, traffic conditions, individual driving styles, and Practical implementation of infrastructure creation. An experiment was conducted on a Nissan LEAF automobile to evaluate its performance under normal driving conditions on Beijing roads, including traffic conditions [18].

A design methodology is suggested for a three-wheeled light electric vehicle with an efficient power distribution system for an electric energy storage system such as a lithium-ion battery or ultra-capacitor [19]. Various factors, such as traffic, terrain, resistive forces, vehicle characteristics, and driving habits, Affect the energy consumption of electric cars (EVs). An EV's range is directly affected by its battery capacity, which is a key factor. The author examines the performance of batteries in electric vehicles (EVs) under several conditions, including urban and highway settings [20].

A theoretical study was conducted to examine the effect of speed and acceleration on energy consumption for specific driving cycles [21]. An accurate MATLAB/Simulink model, including the powertrain system and longitudinal vehicle dynamics, is built to calculate the expected electric car energy consumption [22].

The author of the text focused on studying internal factors such as the battery and motor of a vehicle. The motor has a major impact on the motor range of the vehicle, and a long driving range can be achieved by using batteries with high energy density. Internal and external variables, such as road conditions, can affect a vehicle's range. However, environmental factors must be identified to accurately estimate driving range, as driving conditions are dynamic and ever-changing. To predict accurate results, the author used a combination of Kernel principal component and parameter for fuzzy C clustering [23]. A method has been developed to construct an urban drive cycle (UDC) to study EC. However, this UDC shows a significant error when compared to the international driving cycle. Therefore, to obtain accurate results, the author recommends developing a separate drive cycle for each city and region [24].

The author explains the importance of the driving cycle in ensuring an accurate estimate of the energy consumption of an electric vehicle (EV). The importance of real-world drive cycle design is emphasized in wheel battery management for EV powertrain systems and battery range calculation. As previously mentioned, there is a difference in the result between the simulated drive cycle and the actual driving conditions [25]. A new method for estimating electric vehicle (EV) energy consumption based on Indian driving conditions has been presented. As drive-cycle design may vary between provinces due to variables such as traffic congestion and road conditions, research on precise drive-cycle design has focused exclusively on a limited region (Delhi-Noida). The primary objective is to compare the actual drive cycle with the standardized one, specifically focusing on route selection, data collection, data classification, and drive cycle development [26]. This study analyzes the vehicle dynamics characteristics of a four-wheeler TATA Nexon EV and performs calculations using harnessed DCs (Direct Currents) to determine the range of the vehicle. The range was estimated to be 330 km using NEDC and 288 km using IDC. Highlighting the need for accurately estimating or computing energy consumption to determine a vehicle's driving distance. The study concluded that an electric vehicle with the same specs uses less energy when evaluated using the New European Driving Cycle (NEDC) than the International Driving Cycle (IDC) [27]. An electric two-wheeler scooter was compared with conventional motorcycles to estimate range and power consumption. Research has concluded that the NEDC drive cycle has a range of 130 km, while the IDC gives a range of around 95 km. The author also highlights the need for a strong and resilient road infrastructure in India to optimize the driving range of an electric vehicle [28].

Electric vehicles (EVs) must consume energy accurately and efficiently. The battery capacity, chemistry, size, and weight of an EV depend on the driving range, which in turn relies on the amount of energy consumed per kilometer. Several researchers have developed driving cycles to estimate energy consumption. Some studies have examined how external and internal factors influence the energy consumption of vehicles, such as driving conditions, driver behavior, and road type.

This research is unique because it compares several driving cycles to determine the most efficient performance of four-wheel electric cars and their energy consumption. Five different drive cycles, including WHFET, IDC, NEDC, UDC, WLTP, and Modified Indian Drive Cycle (MIDC), were used in this research. This study comprehensively examines the vehicle's energy consumption per kilometer using the MIDC driving cycle, a hitherto unexplored research area. This research comprehensively examines five driving cycles, unlike previous studies where only two were evaluated. It aims to establish the energy consumption per kilometer for electric vehicles. The article examines the energy consumption of several drive cycle applications to assess their impact on the energy consumption of electric vehicles.

There are significant differences in cycles based on topology and area. This article aims to assist in predicting the precise EC per kilometer of a vehicle, which in turn aids in selecting the correct battery size for four-wheeler applications and determining the expected vehicle range. The article emphasizes the significance of the drive cycle in estimating electrical requirements and sizing the battery for electric vehicles.

The current study aims to select the appropriate drive cycle for accurately estimating the energy consumption of four-wheeler vehicles. Although different drive cycles have been used, there is no standard methodology for choosing the drive cycle for a specific application. Some researchers have developed drive cycles based on the area, city, and region and have demonstrated their accuracy compared to the standard drive cycle.

The researchers have not yet used an analytical approach to analyze the impact of different drive cycles on the EC of electric four-wheeler vehicles. Therefore, this study investigates the EC estimation of electric vehicles using various drive cycles, such as IDC, NEDC, Artemis UDC, MIDC, WLTP, and HWFET. Driving cycles play a critical role in the design and evaluation of EVs.

The main findings of this study are as follows:

• The proposed method provides a higher and lower speed of driving and selection of drive cycle for the highways with a higher range and lower range for the congested cities or rural areas. For the given specifications of an electric vehicle.
• Performance range depends upon the selection of drive cycle and type of road, which will significantly impact the performance range, for higher performance range provided by the WLTP Class-3 drive cycle followed by the NEDC, MIDC, and IDC drive cycles.
• The energy consumption is low for the WLTP Class-3 drive cycle followed by the NEDC, MIDC, and IDC drive cycles, and also slightly it can vary with the selection of drive cycle.
• The state of charge of an EV is also considered; when the drive cycle has more intermittent stages in a particular period, then the SOC of the battery steeply decays, and the state of charge is higher for the lower speed of operation or start-stop condition.
• WLTP Class-3 drive cycle provides for a higher performance range and lower energy consumption, followed by the drive cycles of NEDC, MIDC, and IDC, which are designed for urban road conditions and can withstand all types of road conditions. As per the research, it consumes less power and offers a more extended range than other drive cycles.

The organization of this study is as follows: Section II details the design and considerations for total traction force estimation and validates the mathematical modeling using vehicle dynamics. Section III is the Results and discussion, validates the proposed method and its performance analysis. Finally, Section IV offers the concluding remarks.

## 2 Mathematical modelling of vehicle dynamics for tractive force estimation

### 2.1 Total tractive force estimation

**Fig. 3:**
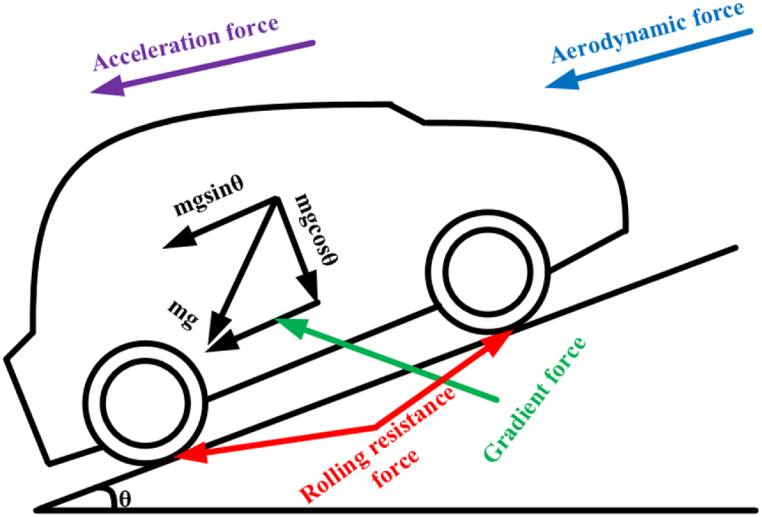
Total Tractive Force estimation.

To calculate the energy consumption of a four-wheeled electric vehicle, the analytical method is used. Estimating the total tractive force exerted on the vehicle is necessary to achieve this goal. Fig. 3 illustrates the many forces contributing to a vehicle's motion, including rolling resistance, aerodynamic resistance, gradient, and acceleration. Determination of these forces is accomplished by using the governing equation of motion. The required power to the motor for acceleration and energy consumption of the vehicle is obtained from Equations (6), (7)). Energy consumption is calculated by combining tractive force values with speed and timing data from the drive wheel. This research assumes that the road's gradient is zero and no forces are acting on the sloping roads.

Rolling resistance force $\left(F_{r}\right)\text{:}$

(1) $$F_{r}=C_{rr}\;mg\;\cos\;\alpha$$

Aerodynamic Force $\left(F_{ad}\right)\text{:}$

(2) $$F_{ad}=\frac{1}{2}\rho C_{d}A\;V^{2}$$

Gradient Force $\left(F_{g}\right)\text{:}$

(3) $$F_{g}=mgsin\alpha$$

Acceleration Force $\left(F_{a}\right)\text{:}$

(4) $$F_{a}=ma$$

Total Traction Force $\left(F_{t}\right)\text{:}$

(5) $$F_{t}=F_{r}+F_{ad}+F_{g}+F_{a}$$

Wheel Power (P):

(6) $$P=F_{t}\times V$$

Energy Consumption $\left(E_{c}\right)\text{:}$

(7) $$E_{c}=P\times t$$

EV Range :

(8) $$Range=\frac{Battery\;capcity\;\left(Wh\right)}{Energy\;consumption\;\left(\frac{Wh}{km}\right)}$$

### 2.2 Drive cycle-based analysis for range and state-of-charge

**Fig. 4:**
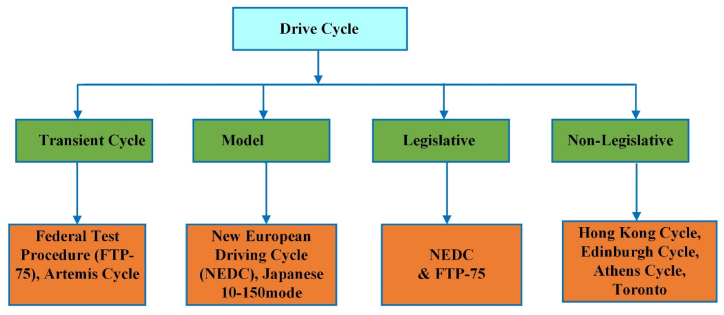
Various types of drive cycles.

A driving cycle is a compilation of vehicle velocities graphed over time. Driving cycles are specifically created to assess the fuel economy (EC), emission of pollutants, and distance covered by vehicles. This assessment applies to both traditional and electric automobiles. Drive cycles universally apply to all vehicles, irrespective of their working conditions. There are two categories of wheels used transient and prototypes globally. A model cycle refers to data collection during constant acceleration and velocity periods. Data may not accurately reflect actual driving behavior. In contrast, temporary wheels have many speed changes that are typical of on-road driving conditions. Legislative and non-legislative drive cycles belong to a distinct group of drive cycles. Regulatory agencies use the statutory driving cycle to verify fuel efficiency and emissions. Non-legislative driving cycles are used in many domains of study, including vehicle design and vehicle life analysis. Shown in Fig. 4 [25].

Several popular and widely used drive cycles exist, including WLTP, NEDC, FTP 75, HWFET, and UDC [25]. Drive cycles are an essential aspect of designing electric vehicles (EVs) and determining the sizes of their components. Vehicle designers and manufacturers use drive cycles to predict how a car will behave during the design stage for both conventional vehicles and EVs. Drive cycles help model the powertrain, sizing components and batteries, estimating energy consumption, and improving vehicle performance. They also help to design transmission systems with reduced costs and minimize the time required for on-road testing. The design of the drive cycle varies by region due to factors such as road types, traffic conditions, and vehicle types [29]. The study aims to identify the best standard drive cycle for accurately sizing the battery and estimating energy consumption in electric vehicles. Four different cycles were used for this purpose.

This research has considered the specifications of the TATA Nexon EV model with the various drive cycles. It consists of four driving cycles (NEDC, MIDC, WLTP Class 3, and IDC) to better compare the results. Table 1 shows the specifications of the Tata Nexon EV model utilized in the study, and the obtained results accurately estimated and evaluated the energy consumption and performance range of an EV.

**Table 1:** The specifications of the whole FWD EV.

**Specifications**	**Value**
Vehicle mass, m (kg)	1470
Wind velocity, $V_{w}\;\left(\frac{m}{s^{2}}\right)$	9.81
Frontal area, (m)	2.9141
Coefficient of rolling resistance, $C_{rr}$	0.015
Air density, $\rho \left(\frac{kg}{m^{3}}\right)$	1.2
Coefficient of Drag, $C_{d}$	0.18
The radius of the wheel, r (m)	0.4612
Road inclination, θ	0

#### 2.2.1 New European drive cycle (NEDC)

**Fig. 5:**
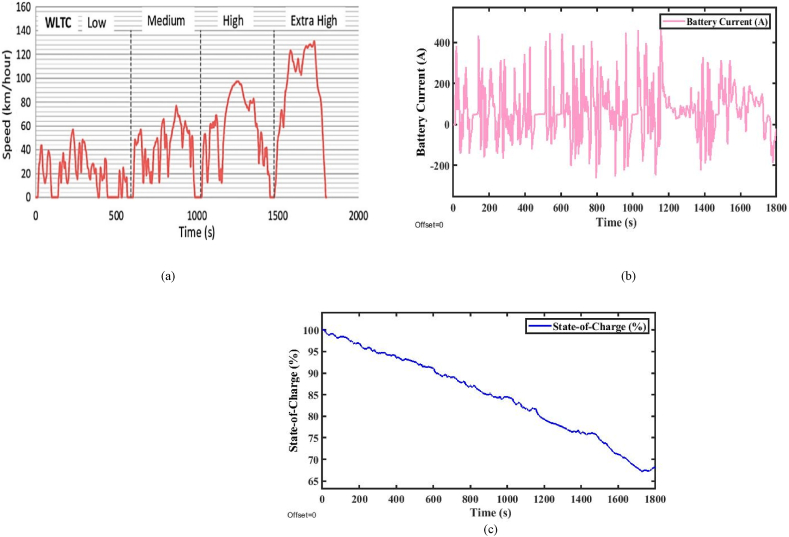
(a). WLTP Class-3 drive cycle Fig. 5(b). Battery current. Fig. 5(c). State-of-Charge (%).

The NEDC is a reference cycle used to estimate vehicle emissions for the Euro VI norm in Europe and some other countries. It comprises an urban and an extra-urban part and was first developed for petrol and diesel cars but can now also be used for EVs to calculate their EC and range. The NEDC drive cycle covers a total distance of 11 km, with an average speed of 33.6 km/h and a total test cycle time of 1180 s. Fig. 5 shows the speed profile of the drive cycle [30].

The parameters of the vehicle are taken from Table 1. We analyze the time-speed data obtained from the NEDC driving cycle to calculate total tractive forces. The rolling resistance force remains constant throughout the analysis as it is not dependent on time or speed. However, the aerodynamic forces are directly proportional to velocity and will vary accordingly.

The force estimation is calculated using Equations (1), (2), (3), (4), (5)), Incorporated with Equations (6), (7)) are utilized to estimate the vehicle's power and ECs. Total energy consumption divided by driving wheel distance. This analysis was performed when reproducibility was assumed to be 100%.

#### 2.2.2 Worldwide harmonized light vehicles test procedure (WLTP Class-3)

Test cycles for chassis dynamo meters, sometimes called internationally standardized light vehicle test cycles, are used to determine the fuel consumption of light-duty vehicles [21,29]. Light vehicles tested in the Worldwide Harmonized Light Vehicle Test Procedure (WLTP) must meet international standards to simulate real-world driving conditions. This includes countries worldwide, including Japan, Korea and India [29]. Three types of this drive cycle are distinguished by the vehicle's maximum speed and power-to-mass ratio (PMR). PMR is the power-to-weight ratio, which is the engine or motor's rated power divided by the vehicle's curb mass.

WLTP classes are classified as given below [21,29].

A. 4. Vehicles in Class 1 are those that have a PMR (Power-to-Mass Ratio) value of 22 or less and a maximum speed of 70 km/h.
B. 4. Class 2 vehicles are defined with a power-to-mass ratio (PMR) between 22 and 34 at a 90 km/h speed or less.
C. 4. Vehicles that can reach 120 km/h or faster speeds and have a power-to-mass ratio (PMR) higher than 34 are categorized as Class 3 type of vehicles.

For a more in-depth study, we focus on the Class 1 cycle, where the vehicle is rated with a PMR of 22. Class 1 refers to a specific group of cars frequently used in India. There are low and medium levels of speed. The duration of one complete cycle of Class 1 is 1022 s, during which the vehicle covers a distance of 8.09 km. The average speed of the car is 64.4 km per hour. This procedure calculates the vehicle's energy consumption per kilometer (EC per km) using the Worldwide Harmonized Light Vehicles Test Procedure (WLTP) cycle.

#### 2.2.3 Modified Indian drive cycle (MIDC)

The Automotive Research Association of India developed the Indian Drive Cycle (IDC) in 1985 to control car emissions in India. The cycle has six different driving modes and lasts 108 s. However, it does not cover all driving conditions found on Indian roads. The average speed at the wheel is 21.9 km/h, which makes it worthwhile to estimate emissions for vehicles with two or three-wheel vehicles [29]. MIDC is an estimation method for calculating pollutant emissions from automobiles and small commercial vehicles. Adoption of the process took place in the year 2000 and was later refined with a more effective cold-start testing method. The MIDC cycle, similar to Europe's NEDC, has been tweaked to better suit Indian conditions by reducing the top speed to 90 kmph. The test was specially conducted to assess the performance of India's BS-IV four-wheeler.

The test duration is 1180 s. The distance covered is 10.7 km, and the average wheel speed is 90 km per hour. These readings calculate the vehicle's energy consumption per kilometer using the MIDC drive cycle approach [29].

#### 2.2.4 Indian drive cycle (IDC)

**Fig. 6:**
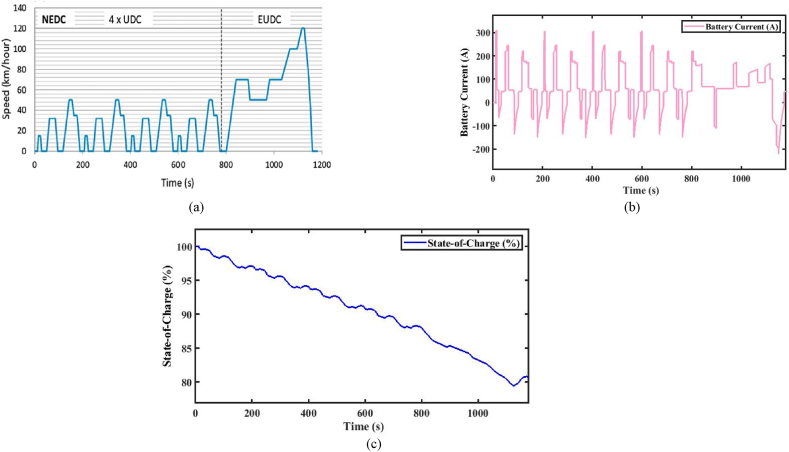
(a). NEDC drive cycle [32] Fig. 6(b). Battery current. Fig. 6(c). State-of-Charge (%).

The Indian Drive Cycle (IDC) has been developed to suit the specific road conditions in India, considering several factors that affect vehicle performance and reliability. An IDC for a two-wheeler consists of six test cycles of 648 s duration. These wheels cover a total distance of 3.948 km. The vehicle reaches a maximum speed of 42 km/h. Fig. 6 provides a graphical depiction of the driving cycle, including speed, acceleration, and duration data points. It also accounts for the hill climb between 59 and 66 s. Based on the vehicle characteristics presented in Table 1, the forces are calculated based on the driving cycle's time and speed data points.

## 3 Simulation results and discussion

### 3.1 Performance range analysis

This article presents a comparative analysis of various drive cycles with the effect of battery current and SoC (%). The battery current and state of charge mainly influence the energy consumption (EC) and the performance of the EV. The analysis is carried out to improve the performance range and the reduction in the energy consumption of EVs by appropriate selection of drive cycles, which are WLTP Class-3, NEDC, MIDC, and IDC, respectively.

### 3.2 WLTP Class-3 drive cycle

Fig. 5(a) represents the WLTP Class-3 (Worldwide Harmonized Light Vehicles Test Procedure) drive cycle. The WLTP Class-3 is a specific test cycle used for electric vehicles. Further, the test-measured parameters include energy consumption and range in a controlled environment. The performance analysis is done by the total force acting on the vehicle specifications given in Table 1. However, the total energy consumption and the range of the vehicle are estimated from Equations (1), (2), (3), (4), (5), (6), (7), (8)) and expressed in Table 2, Table 3 [31]. The corresponding battery current consumed by the vehicle during the analysis of the test and SoC (state-of-charge) % is shown in Fig. 5(b) and (c).

**Table 2:** Energy consumption Comparison of FWD EV with various drive cycles [31].

**Type of Drive Cycle**	**Proposed method (Wh/km)**	**Existed method (Wh/km)**
WlTP Class-3	68.18	96.77
NEDC	80.75	87.56
MIDC	86.41	106.68
IDC	98.51	105.50

**Table 3:** Performance range comparison of FWD EV with various drive cycles [31].

**Type of Drive Cycle**	Proposed method (Km/h)	Existed method (km/h)
WlTP Class-3	135.1	103.33
NEDC	123.8	114.20
MIDC	115.7	93.73
IDC	101.5	93.70

The energy availability at the end of the drive cycle is maintained optimum with the WLTP Class-3 drive cycle because the state of charge at the end of the drive cycle is 65\%. So, the optimum SOC is possible by the WLTP Class-3 drive cycle. An efficient energy saving has been done by the WLTP Class-3 drive cycle. The effect of battery degradation is important in improving the battery's lifetime by maintaining the optimum value of SOC achieved by utilizing the WLTP Class-3 drive cycle. Further, the acceptance of regenerative braking efficiency is greater because the SOC level is optimum in this condition. Then, by the range, improvement has been made throughout the journey. However, the SOC is also affected based on certain parameters considered in practical terrain, speed, and climate.

From the above analysis, It can be concluded that if the driver demands that the required speed of the vehicle is beyond the above-rated speed, then the vehicle is required to operate at the above-rated speed. It will affect the battery current, as shown in Fig. 5(b), and then it will affect the SoC of the vehicle, as shown in Fig. 5(c), due to the back emf of the motor. When the engine is operated at the above-rated speed, in a short time, the power offered by the motor is very high, and with more than 60 % rated power between the time, t = 1500–1800 s with this, the SoC of the vehicle steeply declined. In this analysis, at the end of the drive cycle, the SoC of the car is achieved at about 65 %. The vehicle range offered by the WLTP Class-3 drive-cycle provides a range of 135.1 km/h.

### 3.3 New European Drive Cycle (NEDC)

Fig. 6(a), illustrates the NEDC(New European Drive Cycle). NEDC is a specific test cycle used for electric vehicles. Further, the test-measured parameters include energy consumption and range in a controlled environment. The performance analysis is done by the total force acting on the vehicle specifications given in Table 1. However, the total energy consumption and the range of the vehicle are estimated from Equations (1), (2), (3), (4), (5), (6), (7), (8)) and expressed in Table 2, Table 3 [31]. The corresponding battery current consumed by the vehicle during the analysis of the test and SOC (%) is shown in Fig. 6(b) and (c).

From the above analysis, It can be concluded that if the driver demands that the required speed of the vehicle is beyond the above-rated speed, then the vehicle is required to operate at the above-rated speed. It will affect the battery current, as shown in Fig. 6(b), and then it will affect the SoC of the vehicle, as shown in Fig. 6(c), due to the back emf of the motor. When the engine is operated at the above-rated speed, in a short time, the power offered by the motor is very high. The vehicle's top speed is reached, and there are many identical speeds till the time t = 800 s. In this analysis, at the end of the drive cycle, the SoC of the vehicle is maintained at about 80 % because the peak operating point is not only once, and there is an indication of regeneration. The vehicle range offered by the NEDC drive-cycle provides a range of 123.8 km/h.

### 3.4 Modified Indian Drive Cycle (MIDC)

**Fig. 7:**
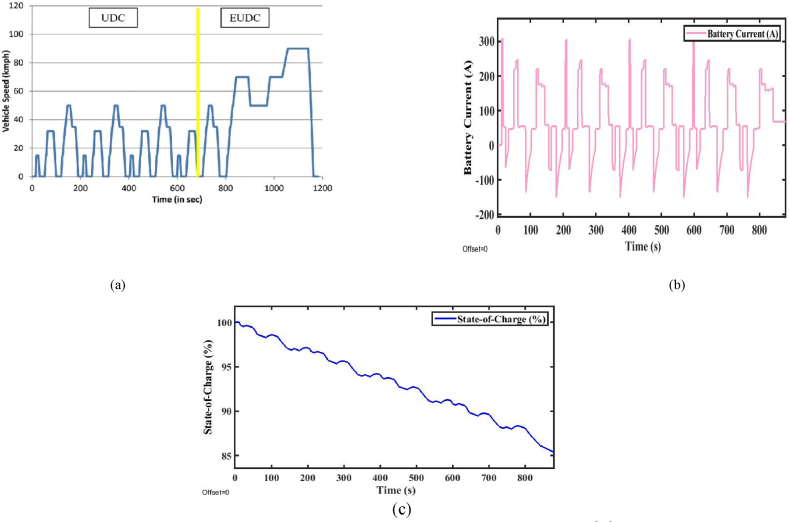
(a). MIDC drive cycle Fig. 7(b). Battery current. Fig. 7(c). State-of-Charge (%).

Fig. 7(a), illustrates the MIDC (Modified Indian Drive Cycle). MIDC is a specific test cycle used for electric vehicles. Further, the test-measured parameters include energy consumption and range in a controlled environment. The performance analysis is done by the total force acting on the vehicle specifications given in Table 1. However, the total energy consumption and the range of the vehicle are estimated from Equations (1), (2), (3), (4), (5), (6), (7), (8)) and expressed in Table 2, Table 3 [31]. The corresponding battery current consumed by the vehicle during the analysis of the test and SOC (%) are shown in Fig. 7(b) and 7(c).

From the above analysis, It can be concluded that if the driver demands that the required speed of the vehicle is beyond the above-rated speed, then the vehicle is required to operate at the above-rated speed. It will affect the battery current, as shown in Fig. 7(b), and then it will affect the SoC of the vehicle, as shown in Fig. 7(c), due to the back emf of the motor. When the engine is operated at the above-rated speed, in a short time, the power offered by the motor is very high. The MIDC-drive cycle is suitable for urban/suburban areas because there is a lot of retardation, and it's primarily used in cities and highways. In this analysis, at the end of the drive cycle, the SOC of the vehicle is maintained at about 85 %. The vehicle range offered by the MIDC drive-cycle provides a range of 115.7 km/h.

### 3.5 Indian Drive Cycle (IDC)

**Fig. 8:**
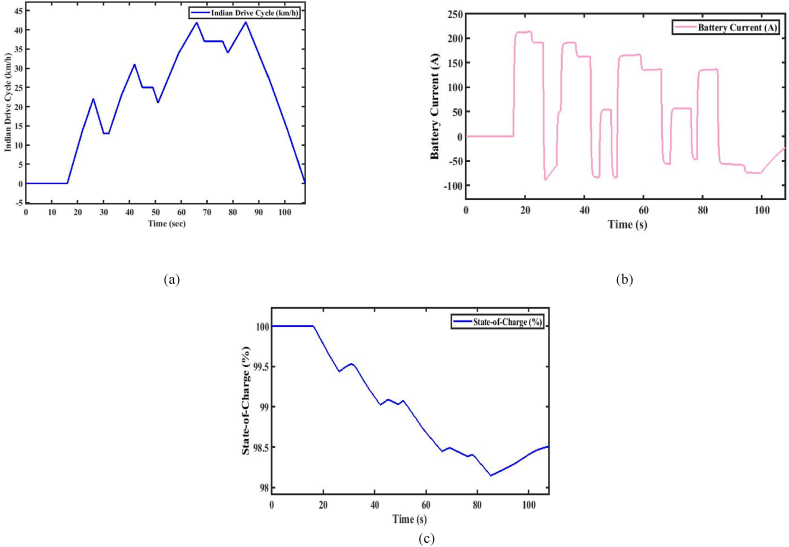
(a). MIDC drive cycle Fig. 8(b). Battery current. Fig. 8(c). State-of-Charge (%)

Fig. 8(a) illustrates the IDC (Indian Drive Cycle). A specific IDC test cycle is used for electric vehicles. Further, the parameters such as energy consumption and range in a controlled environment are tested and measured. The performance analysis is done by the total force acting on the vehicle specifications given in Table 1. However, the total energy consumption and the range of the vehicle are estimated from Equations (1), (2), (3), (4), (5), (6), (7), (8)) and expressed in Table 2, Table 3 [31]. The corresponding battery current consumed by the vehicle during the analysis of the test and SoC (state-of-charge) (%) are shown in Fig. 8(b) and (c).

From the above analysis, it can be concluded that if the driver demands that the required speed of the vehicle is beyond the above-rated speed, then the vehicle is required to operate at the above-rated speed. It will affect the battery current, as shown in Fig. 8(b), and then it will affect the SoC of the vehicle, as shown in Fig. 8(c), due to the back emf of the motor. When the engine is operated at the above-rated speed, in a short time, the power offered by the motor is very high. The IDC-drive cycle is suitable for cities or rural areas because there are a lot of retardation and start-stop operations, and it's primarily used where the traffic is high. In this analysis, at the end of the drive cycle, the SoC of the vehicle is maintained at about 98.5 (%). The vehicle range offered by the IDC drive-cycle provides a range of 101.5 km/h.

In this analysis, the energy consumption and the performance range are obtained from the total capacity of the Li-ion battery. The performance range of EVs mainly depends upon selecting the drive cycle and increasing the total capacity of the lithium-ion battery. This article compares various drive cycles to accurately predict the energy consumption (EC) and range of four-wheeler vehicles. The study shows that the EC per kilometer for different drive cycles, such as WLTP, NEDC, MIDC, and IDC, are illustrated in Table 2. This analysis accurately estimated the energy consumption of an electric vehicle. The study shows that the performance range for different drive cycles, such as WLTP, NEDC, MIDC, and IDC, are illustrated in Table 3. The total capacity of the Li-ion battery is 10,000 (Wh). This study conducted a comparative analysis of several driving cycles to accurately estimate a four-wheeler vehicle's energy consumption (EC) and range. Based on the study conducted, the EC per kilometer for the WLTP Class-3, NEDC, MIDC, and IDC driving cycle was 68.18, 80.75, 86.41, and 98.51, respectively.

**Fig. 9:**
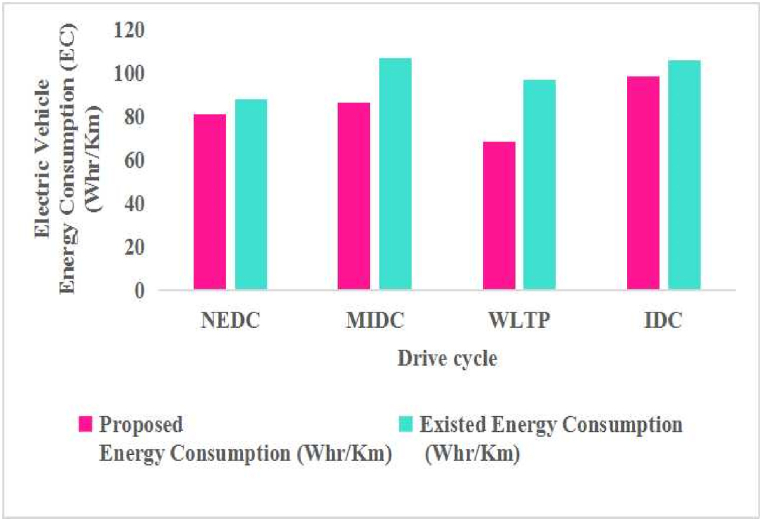
Effect of Energy Consumption with various drive cycles.

**Fig. 10:**
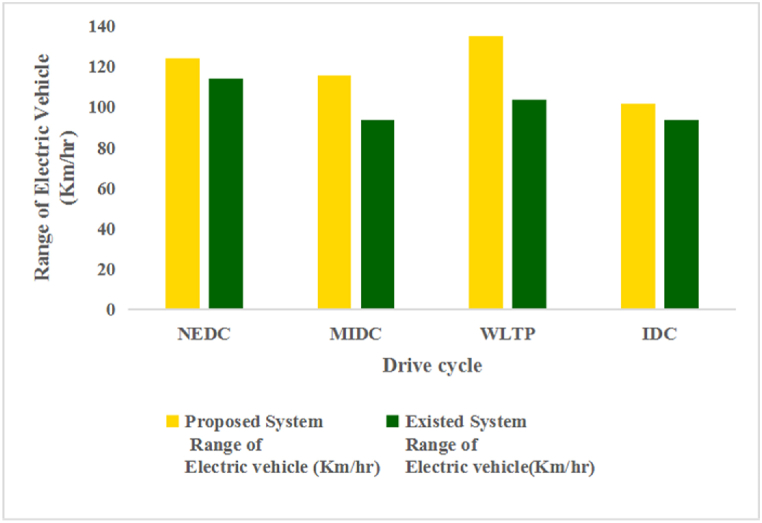
Effect of Range with various drive cycles.

The EC and performance range of an electric vehicle tested in the MATLAB/SIMULINK environment and the obtained result are expressed in Table 2 [31]. For energy consumption of the vehicle, and the range of an electric vehicle is expressed in Table 3 [31]. Based on the above analysis, the energy consumption and the range of an electric vehicle plotted, which is shown in Fig. 9, WLTP Class-3, MIDC, NEDC, and IDC are the various drive cycles are utilized to obtain an accurate estimation of energy consumption for the given specifications. Which is shown in Fig. 10, represents the range of an electric vehicle of various drive cycles such as WLTP Class-3, NEDC, MIDC, and IDC for the given specifications. From the above Figures, Fig. 9 represents the EV energy consumption for the given specifications of the vehicle in Table 1. Electric vehicle energy consumption Wh/km is compared in Table 2. The obtained results show that the EC is lower for the WLTP Class-3, NEDC drive cycles rather than the MIDC and IDC drive cycles, which played mostly on rural road drive cycles because consumption of energy is high for the rural roads rather than the Urban or Extra urban drive cycles, which is shown in Fig. 9.

From the above Figures, Fig. 10 represents the EV Range for the given specifications of the vehicle in Table 1. A comparison was made based on the range obtained for km/hr. The obtained results show that the Range is higher for the WLTP Class-3, NEDC drive cycles rather than the MIDC and IDC drive cycles, which gives a higher range for the Highway, Extra urban, and urban drive cycles rather than the urban or rural road drive cycles because of type of road and the forces acting on the vehicle affects the range of an electric vehicle for various drive cycles.

**Fig. 11:**
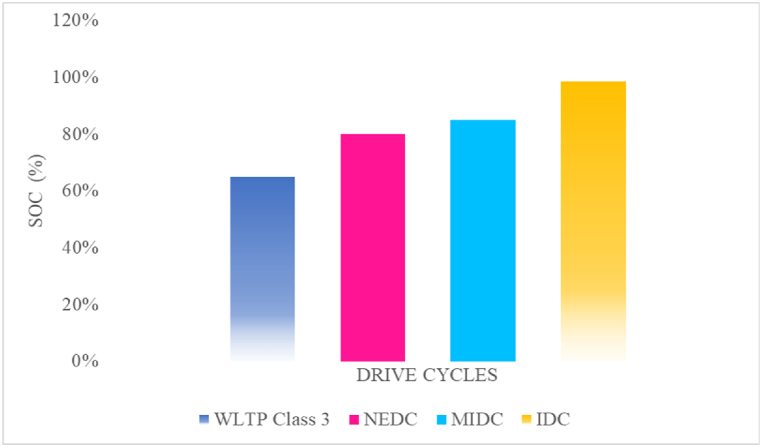
State-of-Charge at the end of a drive cycle.

The State-of-charge analysis claimed for the long drives is much less compared to the driving distance short, because the peak power used by the motor is used many times in this situation and so the State-of-charge affects the vehicle for long-distance driving when compared to the short driving distances. The range offered by the vehicle is more than the short driving distance. Which is shown in Fig. 11.

The selection of the drive cycle plays an important role in the state of charge of an electric vehicle. The study shows that drive cycle selection will also impact the state of charge of an electric vehicle, which is incorporated with the range and EC of EVs. The energy consumption of the battery (Wh/km) and the battery's total capacity give the range of an electric vehicle. In this analysis, a 10,000 Wh Li-ion battery is considered to have a total capacity. The ranges obtained from the aforesaid driving cycles are given by 135.1, 123.8, 115.7, and 101.5 km, respectively. For the above analysis, initially, the state-of-charge is considered 100 (%), and at the end of the drive cycles, it is low depending upon the drive cycle performance, as shown in the Figures from Fig. 5, Fig. 6, Fig. 7, Fig. 8. From the above analysis, it is observed that the state of charge of an EV is steeply declining for the highways and urban drive cycles. Due to the regeneration, the performance range of the vehicle is very high on highways rather than the rural drive cycles, and it also maintains the state of charge for a particular period. SOC also depends upon selecting the appropriate driving cycle, type of road, and driver's driving behavior.

The results demonstrated that the drive cycle selection significantly impacts the EC, and the vehicle performance range can be improved by selecting the optimal parameters of the vehicle dynamics and accurate data driven by the standard drive cycles. Various factors can affect a vehicle's energy consumption (EC), such as the type of climatic conditions, traffic, vehicle weight, drag coefficient, and wind velocity. The performance range obtained for the given specifications of an electric vehicle given a higher range for the WLTP Class-3 drive cycle, which is followed by the NEDC, MIDC, and IDC drive cycles, and the EC of an electric vehicle is lower, and the performance of the vehicle is high, in this condition, the higher performance range is obtained for the lower consumption of energy of an electric vehicle. In this scenario, the energy consumption is lower for the WLTP Class-3 drive cycle, followed by NEDC, MIDC, and IDC drive cycles. The SOC is also one of the parameters that need to be considered. The SOC is higher for the start-stop operations under low-speed conditions, and it does not affect the SOC for higher-speed operations, especially if the intermittent peak operating points are more within short periods than that condition SOC affects more.

However, from the above analysis for the higher speeds and higher range, WLTP Class-3 is more efficient with the regeneration and followed by the NEDC, MIDC, and IDC for rural lower speed application of an electric vehicle is suitable. This analysis is very useful for the estimation of accurate range and then the energy consumption of an electric vehicle, which will be very useful for the Highway, Extra-Urban, urban or city-congested, and rural areas for the selection of drive cycle, which is more useful for the manufacturers of automotive industries. Higher driving speed offers a higher performance range, which will significantly reduce the energy consumption of an electric vehicle and thereby maintain the state of charge of an EV, especially for particular peak operating points that provide more driving range and offer lower energy consumption for the high ways. This analysis is useful for parameter estimation of electric vehicles and selecting drive cycles for improvement ranges in electric vehicles and reductions in energy consumption. Improving an EV's performance by reducing energy consumption and using the traction motor in peak operating points at the intermittent stages will provide a higher performance range and slightly reduce the state of charge.

### 3.4 State-of-charge analysis

The performance range of an electric vehicle (EV) battery is greatly affected by its state of charge (SOC). Below is a comprehensive analysis of the impacts. Further, the parameter's impact on the performance range based on the state of charge is investigated and plotted in Fig. 11. State of charge (SoC) analysis is a key component in managing battery performance in electric vehicles (EVs) and hybrid electric vehicles (HEVs). Drive cycle-based SoC analysis predicts how the battery charge level changes over different driving conditions and cycles. This process is necessary to optimize battery usage, improve energy efficiency, and ensure battery longevity. Drive cycle-based SoC analysis is critical for optimal management of EV and HEV batteries. It provides insights into battery behavior under various driving conditions, helps extend battery life, increases energy efficiency, and ensures accurate range estimates. The above method is the advanced modeling, simulation, and data analysis technique, engineers and researchers can develop advanced BMS and vehicle control strategies that maximize the benefits of electric mobility.

The factors that are affected mainly by the state of charge energy availability, regenerative braking efficiency, thermal management, battery degradation, driving conditions, and SOC management.

#### 3.4.1 WLTP Class-3 drive cycle

Fig. 5(a) represents the SOC of the WLTP Class-3 (Worldwide Harmonized Light Vehicles Test Procedure) drive cycle. The WLTP Class-3 is a specific test cycle used for electric vehicles. Further, the test-measured parameters include energy consumption and range in a controlled environment. The performance analysis is done by the total force acting on the vehicle specifications given in Table 1. However, the total energy consumption and the range of the vehicle are estimated from Equations (1), (2), (3), (4), (5), (6), (7), (8)) and expressed in Table 2, Table 3 [31]. The corresponding battery current consumed by the vehicle during the analysis of the test and SOC (%) are shown in Figures from Fig. 5(b) and 5(c).

The above analysis concluded that the SOC level is high and low based on the driver's required input. If the SOC level is higher than the optimum value, energy availability, battery voltage, and battery degradation, the maximum range of the EV decreases as the battery ages, reduced regenerative braking efficiency, High thermal stress, and speed, terrain, and climate conditions affect mainly. If the SOC level is lower than the optimum value, energy availability is low, and battery voltage, battery degradation, aging effect, enhancing regenerative braking efficiency due to acceptance of braking energy, Low thermal stress, and speed, terrain, and climate conditions are affected mainly. The above analysis is carried out for the WLTP Class-3 Drive Cycle for the state of charge analysis. The drive cycle consists of 65 (%) of SOC throughout the trip, and the maintained SOC level is optimum. The drive cycle is useful for maximizing the improved performance range of highway driving and long-drive trips.

#### 3.4.2 New European Drive Cycle

Fig. 6(a) represents the NEDC (New European Drive Cycle). NEDC is a specific test cycle used for electric vehicles. Further, the test-measured parameters include energy consumption and range in a controlled environment. The performance analysis is done by the total force acting on the vehicle specifications given in Table 1. However, the total energy consumption and the range of the vehicle are estimated from Equations (1), (2), (3), (4), (5), (6), (7), (8)) and expressed in Table 2, Table 3 [31]. The corresponding battery current consumed by the vehicle during the analysis of the test and SOC (%) are shown in Figures from Fig. 6(b) and 6(c).

The above analysis concluded that the SOC level is high and low based on the driver's required input. If the SOC level is higher than the optimum value, energy availability, battery voltage, and battery degradation, the maximum range of the EV decreases as the battery ages, reduced regenerative braking efficiency, High thermal stress, and speed, terrain, and climate conditions affect mainly. If the SOC level is lower than the optimum value, energy availability is low, and battery voltage, battery degradation, aging effect, enhancing regenerative braking efficiency due to acceptance of braking energy, Low thermal stress, and speed, terrain, and climate conditions are affected mainly. The above analysis is carried out for the NEDC Drive Cycle for the state of charge analysis. The drive cycle consists of an 80 (%) SOC throughout the trip, and the maintained SOC level is optimum. The drive cycle is useful for maximizing the improved performance range of highway driving and long drive trips.

#### 3.4.3 Modified Indian Drive Cycle

Fig. 7(a) represents the MIDC (Modified Indian Drive Cycle). MIDC is a specific test cycle used for electric vehicles. Further, the test-measured parameters include energy consumption and range in a controlled environment. The performance analysis is done by the total force acting on the vehicle specifications given in Table 1. However, the total energy consumption and the range of the vehicle are estimated from Equations (1), (2), (3), (4), (5), (6), (7), (8)) and expressed in Table 2, Table 3 [31]. The corresponding battery current consumed by the vehicle during the analysis of the test and soc are shown in Figures from Fig. 7(b) and (c).

The above analysis concluded that the SOC level is high and low based on the driver's required input. If the SOC level is higher than the optimum value, energy availability, battery voltage, and battery degradation, the maximum range of the EV decreases as the battery ages, reduced regenerative braking efficiency, High thermal stress, and speed, terrain, and climate conditions affect mainly. If the SOC level is higher than the optimum value, energy availability is high, and improved battery voltage, high battery degradation, aging effect, low-performance on regenerative braking efficiency due to acceptance of braking energy, high thermal stress, and speed, terrain, and climate conditions are affected mainly. The above analysis is carried out for the MIDC Drive Cycle for the state of charge analysis. The drive cycle consists of an 85 (%) SOC throughout the trip, and the maintained SOC level is optimum. The drive cycle is useful for maximizing the improved performance range of urban/city road conditions.

#### 3.4.4 Indian Drive Cycle

Fig. 8(a) represents the IDC (Indian Drive Cycle). A specific IDC test cycle is used for electric vehicles. Further, the parameters such as energy consumption and range in a controlled environment are tested and measured. The performance analysis is done by the total force acting on the vehicle specifications given in Table 1. However, the total energy consumption and the range of the vehicle are estimated from Equations (1), (2), (3), (4), (5), (6), (7), (8)) and expressed in Table 2, Table 3 [31]. The corresponding battery current consumed by the vehicle during the analysis of the test and soc is shown in Figures from Fig. 8(b) and c.

The above analysis concluded that the SOC level is high and low based on the driver's required input. If the SOC level is higher than the optimum value, energy availability, battery voltage, and battery degradation, the maximum range of the EV decreases as the battery ages, reduced regenerative braking efficiency, High thermal stress, and speed, terrain, and climate conditions affect mainly. If the SOC level is higher than the optimum value, energy availability is high, and reduced battery voltage, battery degradation, and aging affect low performance on regenerative braking efficiency due to acceptance of braking energy, high thermal stress, and speed, terrain, and climate conditions are affected mainly. The above analysis is carried out for the IDC Drive Cycle for the state of charge analysis. The drive cycle consists of 98.5 (%) SoC throughout the trip, and the maintained SOC level is optimum. The drive cycle maximizes the improved performance range of city-congested driving and start-stop road conditions.

## 4 Conclusion

Electric vehicles (EVs) are becoming more popular because of their emission-free technology, cost-effective operation, and enhanced efficiency. Nevertheless, to enhance the affordability of electric vehicles (EVs) for consumers, it is essential to decrease the cost per kilometer of power utilized and augment the vehicle's range. The cost per km is determined by several elements, including static and dynamic characteristics, and is highly dependent on the driving cycle. Recent research has shown that rural drive cycles yield more energy consumption than other drive cycles, highlighting the need for a more precise calculation of energy consumption. An accurate driving cycle would be beneficial for automotive manufacturers in the development of advanced electric vehicles. This research analyzed the gap in several driving cycles, including WLTP Class-3, NEDC, MIDC, and IDC drive cycles. Range obtained from above drive WLTP Class-3, NEDC, MIDC, and IDC drive cycles 135.1123.8115.7 and 101.5 km, respectively. Due to improved road conditions, the WLTP Class-3 drive cycle offers more than the others. Due to road conditions, the IDC drive cycle offers a shorter range of 101.5 km. These driving cycles accommodated various road conditions, traffic scenarios, vehicle kinds, and global areas. Manufacturers and designers may better understand a vehicle's electrical consumption and range by considering various driving cycles. This study offers vital insights for precisely estimating the energy consumption requirements of a vehicle and choosing an acceptable driving pattern. An effective analysis is done based on the performance range and SOC of an electric vehicle for given test specifications and specified standard drive cycles. The analysis is accurately utilized for EV manufacturers or suppliers and is also suitable for upcoming researchers developing more control strategies in electric vehicle technology using artificial intelligence and machine learning algorithms.

## CRediT authorship contribution statement

**Shana Lakshmi Prasad:** Writing – original draft, Software, Resources, Methodology, Investigation, Data curation. **Abhishek Gudipalli:** Writing – review & editing, Validation, Supervision, Software, Resources, Project administration, Investigation, Formal analysis.

## Data availability statement

The datasets generated and analyzed during the current study are available from the corresponding author upon reasonable request.

## Funding

Not applicable.

## Declaration of competing interest

The authors declare that they have no known competing financial interests or personal relationships that could have appeared to influence the work reported in this paper.

## Nomenclature and Acronyms

A: frontal area of the vehicle
Cd: coefficient of drag
Cr: rolling resistance coefficient
Etotal: total energy consumption
Fa: acceleration force
Fd: aerodynamics force
Fg: gradient force
Fr: rolling resistance force
Ft: total tractive force
m: vehicle gross weight
r: wheel radius
